# A comprehensive comparison of sex-inducing activity in asexual worms of the planarian *Dugesia ryukyuensis*: the crucial sex-inducing substance appears to be present in yolk glands in Tricladida

**DOI:** 10.1186/s40851-018-0096-9

**Published:** 2018-06-12

**Authors:** Haruka Nakagawa, Kiyono Sekii, Takanobu Maezawa, Makoto Kitamura, Soichiro Miyashita, Marina Abukawa, Midori Matsumoto, Kazuya Kobayashi

**Affiliations:** 1 Department of Biology, Faculty of Agriculture and Life Science, Hirosaki University, 3 Bunkyo-cho, Hirosaki, Aomori, 036-8561 Japan; 2 Advanced Science Course, Department of Integrated Science and Technology, National Institute of Technology, Tsuyama College, 624-1 Numa, Tsuyama, Okayama, 708-8509 Japan; 3 Center for Integrated Medical Research, School of Medicine, Keio University, 35 Shinanomachi, Shinjuku-ku, Tokyo, 160-8582 Japan; 4 Department of Biosciences and Informatics, Keio University, 3-14-1 Hiyoshi, Kouhoku-ku, Yokohama, 223-8522 Japan

**Keywords:** Turbellaria, Triclad, Polyclad, Planarian, *Dugesia ryukyuensis*, Asexual reproduction, Sexual reproduction, Sexual induction, Sex-inducing substance

## Abstract

**Background:**

Turbellarian species can post-embryonically produce germ line cells from pluripotent stem cells called neoblasts, which enables some of them to switch between an asexual and a sexual state in response to environmental changes. Certain low-molecular-weight compounds contained in sexually mature animals act as sex-inducing substances that trigger post-embryonic germ cell development in asexual worms of the freshwater planarian *Dugesia ryukyuensis* (Tricladida). These sex-inducing substances may provide clues to the molecular mechanism of this reproductive switch. However, limited information about these sex-inducing substances is available.

**Results:**

Our assay system based on feeding sex-inducing substances to asexual worms of *D. ryukyuensis* is useful for evaluating sex-inducing activity. We used the freshwater planarians *D. ryukyuensis* and *Bdellocephala brunnea* (Tricladida), land planarian *Bipalium nobile* (Tricladida), and marine flatworm *Thysanozoon brocchii* (Polycladida) as sources of the sex-inducing substances. Using an assay system, we showed that the three Tricladida species had sufficient sex-inducing activity to fully induce hermaphroditic reproductive organs in asexual worms of *D. ryukyuensis*. However, the sex-inducing activity of *T. brocchii* was sufficient only to induce a pair of ovaries. We found that yolk glands, which are found in Tricladida but not Polycladida, may contain the sex-inducing substance that can fully sexualize asexual worms of *D. ryukyuensis*.

**Conclusions:**

Our results suggest that within Tricladida, there are one or more common compounds or functional analogs capable of fully sexualizing asexual worms of *D. ryukyuensis*; namely, the crucial sex-inducing substance (hydrophilic and heat-stable, but not a peptide) produced in yolk glands.

**Electronic supplementary material:**

The online version of this article (10.1186/s40851-018-0096-9) contains supplementary material, which is available to authorized users.

## Background

Metazoans occasionally switch their mode of reproduction on the basis of environmental changes, life cycle phase, or both. However, the mechanisms underlying the switch from an asexual to a sexual mode of reproduction and vice versa are poorly understood. Scyphozoan cnidarian, *Aurelia aurita,* seasonally switches their life cycle between asexual polyps and sexual medusae [1]. Under laboratory conditions, the switch from polyp to medusa can be induced by lowering the water temperature. The mechanism controlling the switch consists of retinoic signaling and temperature-sensitive signaling by secreted protein CL390, which encodes the precursor of a putative peptide hormone [1]. The administration of 9-cis-RA or the deduced peptide hormone from CL390 to the polyps (the asexual state) triggers the metamorphosis to the medusa (the sexual state). Therefore, the compounds that control this switch from an asexual to a sexual state will possibly provide clues to help elucidate the molecular mechanism for the reproductive switch. We call such a compound a sex-inducing substance.

Some freshwater planarians (Platyhelminthes, Turbellaria, Tricladida, and Continenticola) can reproduce asexually as well as sexually. Sexual worms have hermaphroditic reproductive organs. In contrast, asexual worms regenerate lost body parts after fission without developing reproductive organs [2]. Therefore, when asexual worms switch to a sexual state, i.e., sexual induction based on environmental stimuli [3–6], they differentiate hermaphroditic reproductive organs from pluripotent stem cells called neoblasts [7–15]. The existence of a planarian sex-inducing substance(s) was suggested by an experimental sexual induction by “feeding” [16–20]. If asexual planarians are fed minced sexually mature worms of the same or different freshwater planarian species, they develop reproductive organs without having been exposed to the environmental stimuli that typically induce this switch (Additional file 1). This suggests that a sex-inducing substance(s) contained in sexually mature worms is a common compound(s) or functional analog(s) in freshwater planarians.

**Fig. 1:**
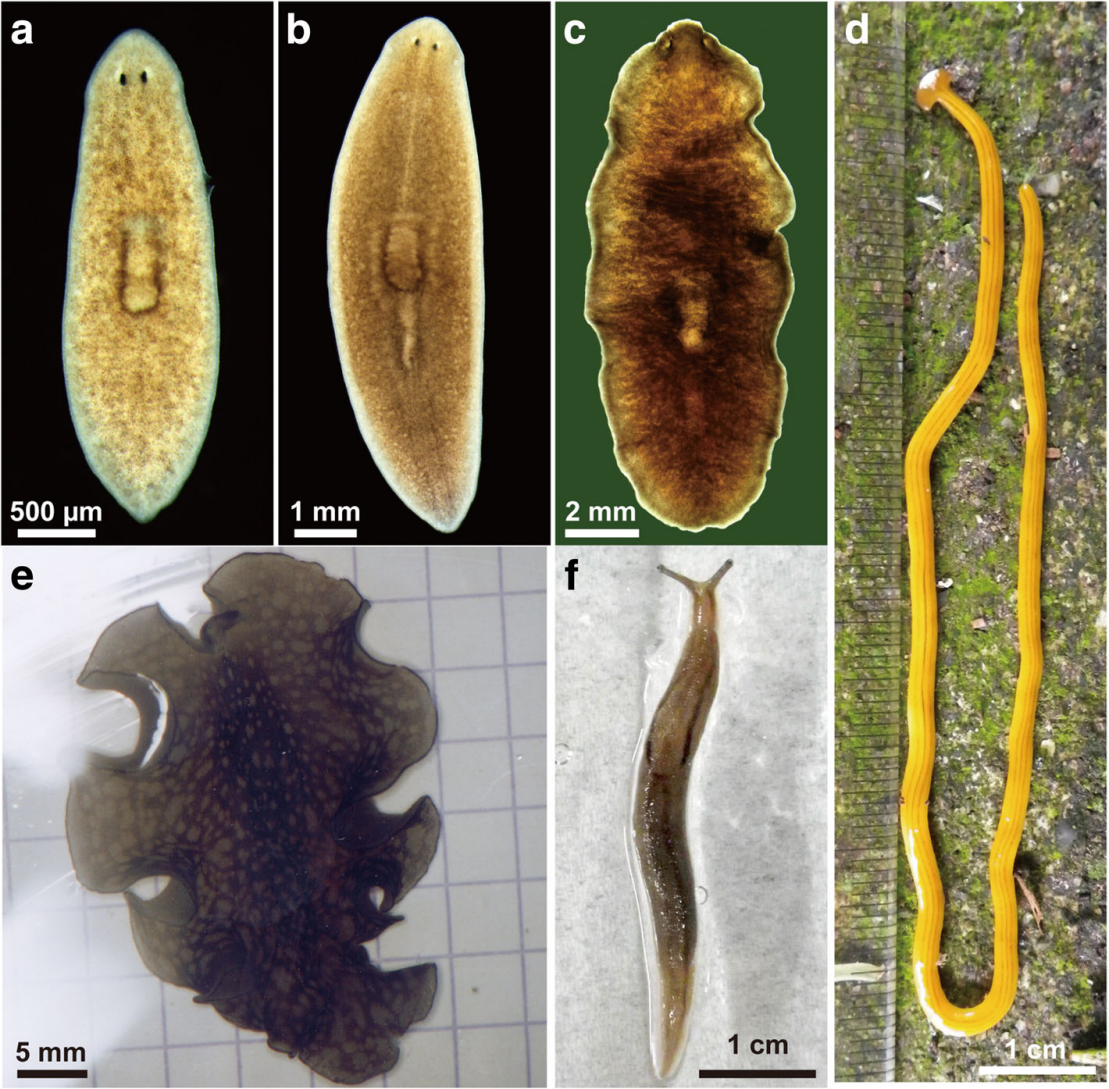
Images of the five species used in this study. **a** The asexual worm (OH strain) of the freshwater planarian *Dugesia ryukyuensis* (Tricladida, Continenticola, Dugesiidae). **b** The sexual worm of *D. ryukyuensis*. **c** The freshwater planarian *Bdellocephala brunnea* (Tricladida, Continenticola, Dendrocoelidae). **d** The land planarian *Bipalium nobile* (Tricladida, Continenticola, Bipaliidae). **e** The marine flatworm *Thysanozoon brocchii* (Polycladida). **f** The slug *Ambigolimax valentianus* (Mollusca)

We established an assay system for isolating the sex-inducing substance(s). Asexual *Dugesia ryukyuensis* of the OH strain (Tricladida, Continenticola, Dugesiidae) were stimulated to develop hermaphroditic reproductive organs by being fed conspecific sexual worms and sexually mature *Bdellocephala brunnea* worms (Tricladida, Continenticola, Dendrocoelidae) (Fig. 1a–c) [21, 22]. Recently, we found that d-Trp is involved in ovarian development of asexual worms as a sex-inducing substance [23]. However, d-Trp does not trigger complete sexual induction in asexual worms. Thus, a crucial sex-inducing substance(s), which is needed for complete sexual induction, has not yet been identified. Since there is no prior evidence whether complete sexual induction can be attributed to a single substance or multiple substances, we refer to the crucial sex-inducing substance(s) in the singular form throughout this paper. Moreover, limited information is available about whether any phylogenetic range of species might contain the crucial sex-inducing substance that can induce reproductive switching in *D. ryukyuensis*. Such information about the range of species with sex-inducing activity toward asexual worms of *D. ryukyuensis* will contribute to the identification of the crucial sex-inducing substance.

Turbellaria comprise two macroturbellarians (Tricladida and Polycladida) and nine microturbellarians [24, 25]. Microturbellarians are not quantitatively suitable as sources of putative sex-inducing substances in our assay system. In this study, to narrow down the phylogenetic range of species with sex-inducing activity toward asexual worms of *D. ryukyuensis*, where possible, we used the land planarian *Bipalium nobile* (Tricladida, Continenticola, Bipaliidae) and marine flatworm *Thysanozoon brocchii* (Polycladida), with *D. ryukyuensis* and *Bd. brunnea* as sources of a sex-inducing substance (Fig. 1d, e). A slug, *Ambigolimax valentianus* (Mollusca), a natural food source for *Bi. nobile*, was also used (Fig. 1f). To examine the potency of their sex-inducing activity toward asexual worms of *D. ryukyuensis*, we compared sex-inducing activity in four fractions from the five species obtained by a fractionation method using this assay system. Here, we report that the crucial sex-inducing substance may be a common compound or functional analog that is produced in the yolk glands in Tricladida.

## Methods

### Animals

An exclusively asexual strain, the OH strain, of the freshwater planarian *D. ryukyuensis* (Fig. 1a) was maintained at 20 °C in dechlorinated tap water and fed chicken liver once a week. Worms of this strain were used as test animals for sexual induction. Sexual worms of *D. ryukyuensis* (Fig. 1b) were obtained by feeding worms of the OH strain with sexual worms as described previously [22]. The sexual worms of *D. ryukyuensis* were cut and allowed to regenerate. They were maintained at 20 °C in dechlorinated tap water and fed chicken liver once a week until maturity. They then began to lay cocoons constantly. The sexually mature worms and the fresh cocoons were collected within a day of deposition were stored at − 80 °C for use as a source of the sex-inducing substance. Sexually mature populations of the freshwater planarian *Bd. brunnea* (Fig. 1c), land planarian *Bi. nobile* (Fig. 1d), marine flatworm *T. brocchii* (Fig. 1e), and slug *A. valentianus* (Fig. 1f) were collected near Yamagata City, Shinjuku-ku, Tokyo, the Misaki Marine Station of Tokyo University, and Chofu City, Tokyo, respectively, in Japan. The fresh cocoons of *Bd. brunnea* were collected within a day of deposition. They were also frozen in liquid nitrogen and stored at − 80 °C for use as a source of the sex-inducing substance.

### Preparation of foods for the bioassay of sexual induction

**Fig. 2:**
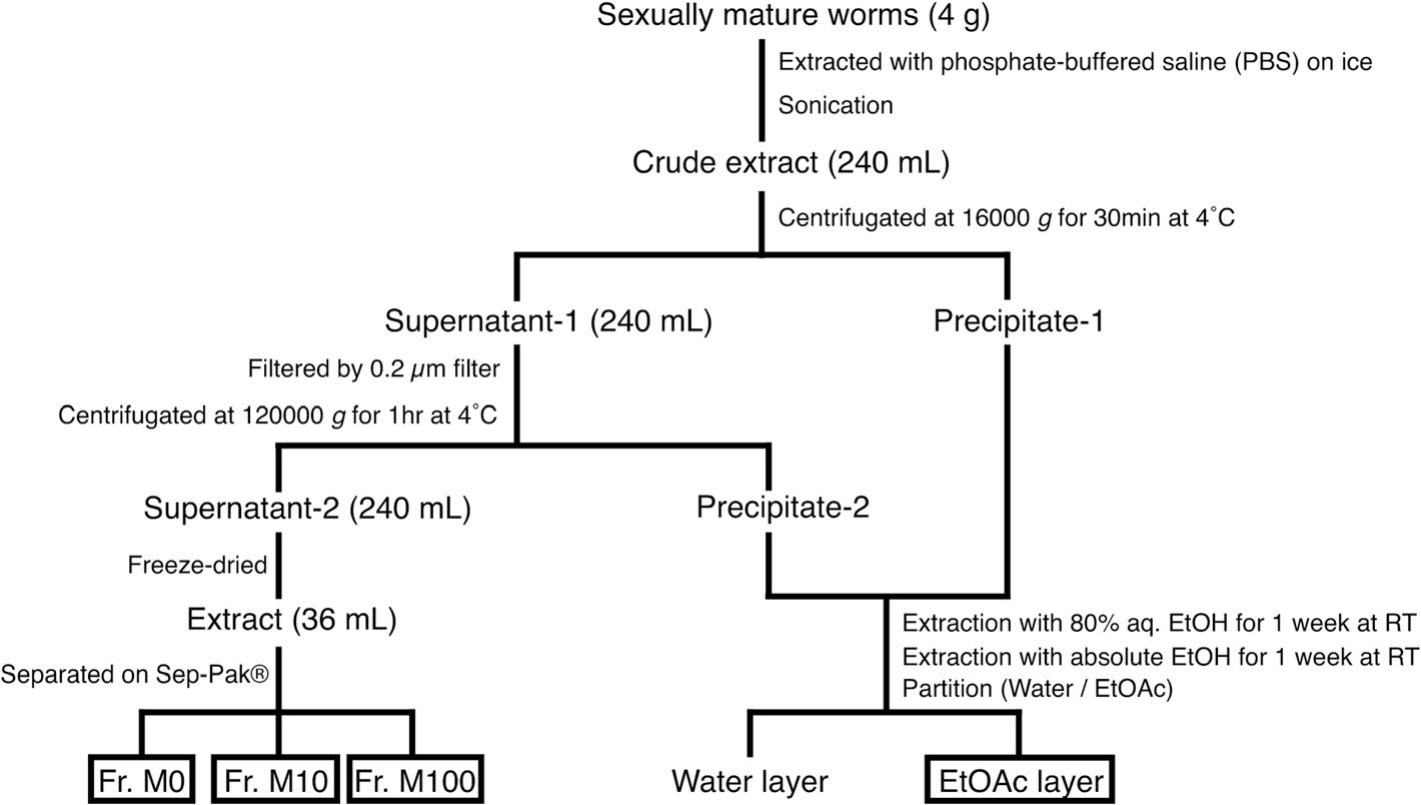
Procedure for the fractionation of sexually mature worms. See the text for experimental details. Fr., fraction. Three fractions and a layer used for the bioassay are highlighted in the box

Figure 2 shows the fractionation procedure for the assay of sex-inducing activity by a method described previously, with a modification [26]. Approximately 4 g wet weight of sexually mature worms of *D. ryukyuensis*, *Bd. brunnea*, *Bi. nobile, T. brocchii*, and *A. valentianus*, respectively, was homogenized in 240 mL of PBS (34 mM NaCl, 0.68 mM KCl, 2.5 mM Na_2_HPO_4_, and 0.45 mM KH_2_PO_4_; pH 7.4). The homogenate was centrifuged at 16000×*g* for 30 min at 4 °C. The supernatant, or cytosolic fraction, was filtrated using a 0.2 μm filter (CORNING, Lowell, MA) and then centrifuged at 120000×*g* for 1 h at 4 °C. The cytosolic fraction was loaded onto a Sep-Pak® Light tC_18_ Cartridge (Waters, Milford, MA) and eluted with 0, 10, and 100% aqueous methanol to create the following fractions: Fr. M0, Fr. M10, and Fr. M100. The dry weight of each fraction was measured (Additional file 2). After two-step centrifugation, the precipitate (Precipitate-1 and -2 in Fig. 2) was extracted with 80% aqueous ethanol for 1 week at 20 °C. The residue was further extracted with absolute ethanol for 1 week at room temperature. The extraction was evaporated in vacuo to yield a residue, which was partitioned between water (50 mL) and ethyl acetate (50 mL) three times. To facilitate better partitioning, 1 g NaCl was added to the partitioned solution. The dry weight of the ethyl acetate layer (EtOAc layer) was measured (Additional file 3).

### Bioassay and estimation of sex-inducing activity

In this study, we set the standard dose of each sample for the bioassay at 3.9 mg dry weight to compare sex-inducing activity. To produce the test food for the bioassay, we mixed 3.9 mg of each dried sample with 200 μL of chicken liver homogenate, which is used as a food for planarian maintenance, and then freeze-dried the mixture. Freeze-dried chicken liver homogenate was used as a negative (vehicle) control. Thirty test worms were fed a piece of food daily for 4 weeks.

**Fig. 3:**
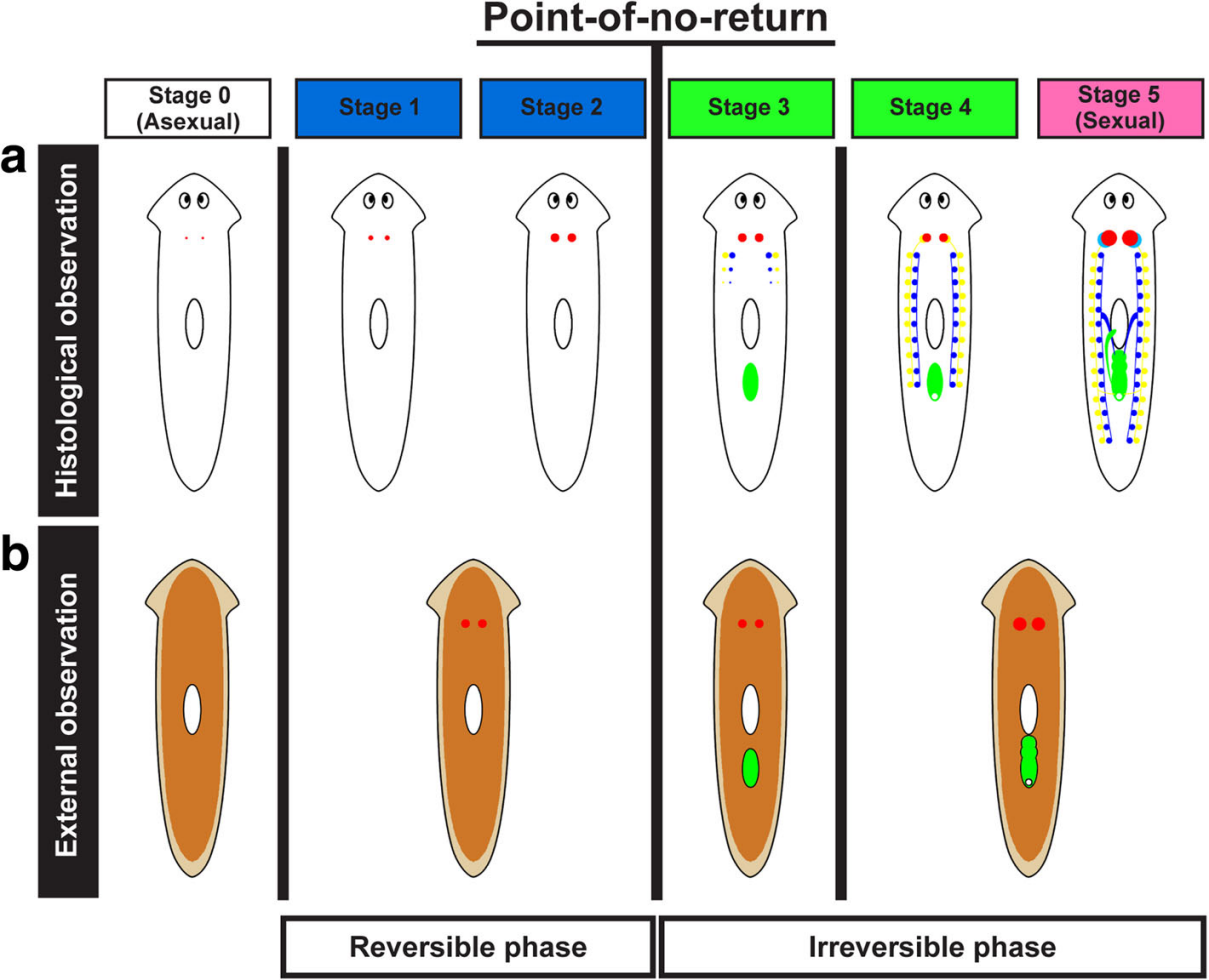
Five stages of sexual induction and estimation of stages by external observation. **a** Morphological changes during sexual induction allowed us to divide the process into five stages. The colored regions correspond to the reproductive organs: red, ovary; aqua blue, seminal receptacle; blue, testis; yellow, yolk gland; green, copulatory apparatus. Cell masses of female primordial germ cells (primordial ovaries) were histologically identified in the asexual worms (stage 0), although they were barely visible externally. The ovaries became sufficiently large to be externally apparent behind the head, although no oocytes or other reproductive organs were detectable at stage 1. Oocytes appeared in the ovaries, but other reproductive organs remained undetectable at stage 2. The primordial testes and yolk gland primordia emerged, and a copulatory apparatus became visible in the post-pharyngeal region at stage 3. The genital pore in the copulatory apparatus opened on the ventral side of the worm and spermatocytes appeared in the testes at stage 4. Mature yolk glands formed, and spermatids and spermatozoa were detectable in the testes at stage 5. **b** By external observation of the test worms, we can recognize a pair of ovaries and a copulatory apparatus (without or with a genital pore) in the ventral side of test worms. The colored regions correspond to these reproductive organs: red, ovary; green, copulatory apparatus. Three stages were identified by external observation: worms with only a pair of ovaries (stage 1–2), worms with a copulatory apparatus without a genital pore (stage 3), and worms with a copulatory apparatus with a genital pore (stage 4–5). This sexual induction has a point-of-no-return between stages 2 and 3. In the reversible phase, worms degenerate a pair of developing ovaries to return to asexual if feeding with sexual worms is stopped. In the irreversible phase, worms continue developing all the reproductive organs, even if feeding with sexual worms is stopped

Each of the test worms developed a pair of ovaries, testes, yolk glands, and a copulatory apparatus in sequence if the test food contained a sufficient quantity and quality of the sex-inducing substance. This morphological change allowed us to divide the sexual induction process into five distinct stages (Fig. 3a) [22]. After the feeding assay, external observations were carried out under a binocular microscope, with specific attention paid to the development of the ovaries, a copulatory apparatus, and a genital pore. By external observation of the test worms, we identified those with only a pair of ovaries (stage 1–2), those with a copulatory apparatus (stage 3), and those with a genital pore (stage 4–5) (Fig. 3b).

This experimental sexual induction has a point-of-no-return between stages 2 and 3. Worms at stages 1 and 2 return to the asexual state if the administration of the sex-inducing substance is stopped, whereas from stages 3 onward worms will continue to develop sexual organs, even if the administration of the sex-inducing substance is stopped, which suggests reversible and irreversible phases as evidenced by the point-of-no-return from external observations (Fig. 3). In the present study, a crucial sex-inducing substance means a compound responsible for overcoming the point-of-no-return.

### Digestion of Fr. M0 and M10 derived from *Bd. brunnea* by Actinase E

Foods were prepared for bioassay of the Fr. M0 and M10 fractions of *Bd. brunnea* treated with Actinase E (KAKEN PHARMACEUTICAL CO., LTD.), which is a powerful enzyme for the elimination of peptides/proteins (Additional file 4). The Fr. M0 and M10 fractions from approximately 8 g wet weight of sexually mature worms of *Bd. brunnea* were prepared according to the fractionation procedure shown in Fig. 2. Actinase E was added to each solution containing Fr. M0 or M10 from approximately 4 g wet weight at a final concentration of 0.1% (*w*/*v* in water). The reaction solutions were incubated at 37 °C for 16 h, and then boiled for 15 min to deactivate Actinase E. As a control (− Actinase E), the solutions containing the Fr. M0 or M10 from approximately 4 g wet weight and 0.1% Actinase E solution were independently incubated and boiled, and finally mixed. To produce the test food for the bioassay, we mixed each dried four sample (Fr. M0 + Actinase E, Fr. M0 – Actinase E, Fr. M10 + Actinase E and Fr. M10 – Actinase E) with 200 μL of chicken liver homogenate, and then freeze-dried the mixture. Thirty test worms were fed a piece of food daily for 4 weeks.

### Histology

Test worms were relaxed in cold 2% (*v*/*v*) HCl in 5/8 Holtfreter’s solution [27] for 5 min and then fixed in 4% paraformaldehyde and 30% ethanol in 5/8 Holtfreter’s solution for 3 h at room temperature. The fixed specimens were dehydrated through an ethanol series, cleared in xylene, and embedded in Paraplast Plus embedding medium (Sigma-Aldrich Co., St. Louis, MO, USA). The embedded specimens were cut into 4 μm thick sections and stained with hematoxylin and eosin.

### Statistical analysis

Data pertaining to the occurrence of worms at stages 1–2, worms from stage 3 onward, and worms at stages 4–5 (Fig. 3b) were analyzed using chi-square or Fisher’s tests.

## Results

### Comparison of sex-inducing activity on asexual *D. ryukyuensis*

According to the fractionation method for the sex-inducing substance [26], we homogenized 4 g of worms in phosphate-buffered saline (PBS) and then obtained the cytosolic fraction of the supernatant (Supernatant-2) and two fractions of the precipitates (Precipitate-1 and -2) after a two-step centrifugation (Fig. 2). Compounds that are more hydrophilic must be extracted into the cytosolic fraction, whereas compounds that are more hydrophobic must be contained in the precipitates. In the present study, each cytosolic fraction from the five species was applied to a commercial octadecylsilane (ODS) column and eluted stepwise by changing the methanol concentration of the eluent (0, 10, and 100% (*v*/*v*)) (Fig. 2 and Additional file 2). Each precipitate was extracted with ethanol. To reliably remove the residual hydrophilic compounds in the precipitates, the extractions were partitioned between water and ethyl acetate. Since 1 g NaCl was added to the partitioned solutions to facilitate better partitioning, the test worms could not eat the water layer owing to a high salt concentration. Consequently, compounds that are more hydrophobic must be recovered in the ethyl acetate layer (EtOAc layer) (Fig. 2 and Additional file 3).

**Fig. 4:**
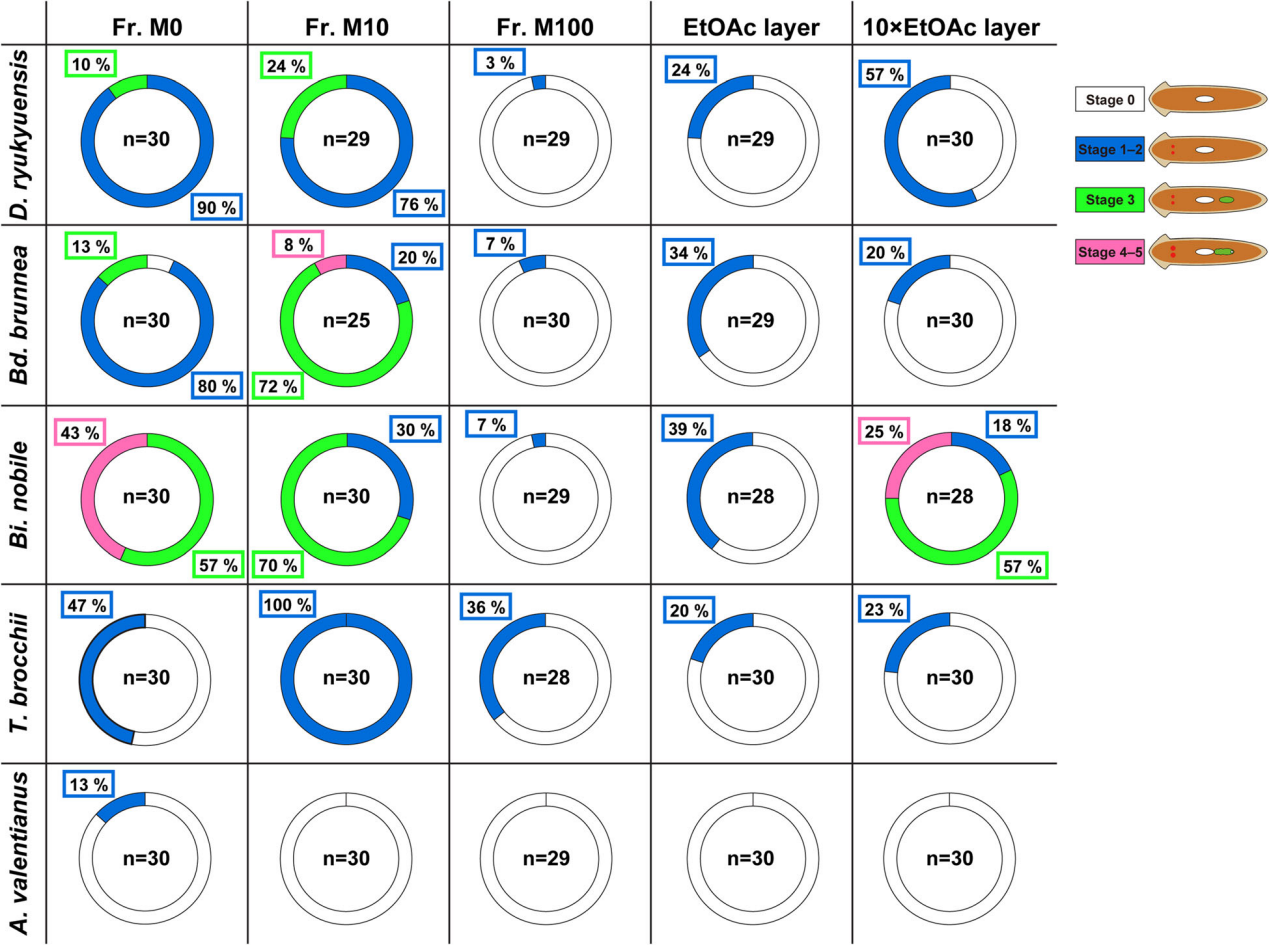
Comparison of sex-inducing activity toward asexual worms of *D. ryukyuensis.* For the assay of the three fractions from the cytosolic fraction and EtOAc layer, test worms at the three stages of sexual induction identified by external observation were expressed as a doughnut chart. The number of test worms after the bioassay is shown in the center of a doughnut chart. The illustrations of worms in the figure correspond to those in Fig. 3b. The percentage of worms at each of the three stages of sexual induction is shown in the box drawn by colored line

In a previous study, fractions of *D. ryukyuensis* and *Bd. brunnea* that had been eluted with water (Fr. M0) and 10% methanol (Fr. M10) exhibited sufficient sex-inducing activity in asexual worms of *D. ryukyuensis* to overcome the point-of-no-return [26]. In the present study, because the fraction with the smallest dry weight (3.9 mg) was Fr. M10 from *Bd. brunnea* (Additional files 2 and 3), which showed the strong sex-inducing activity [26], we set the standard dose of each sample for the bioassay at 3.9 mg dry weight to compare sex-inducing activity. Figure 4 shows sex-inducing activity in the three fractions from the cytosolic fraction and the EtOAc layer toward asexual worms of *D. ryukyuensis*.

**Fig. 5:**
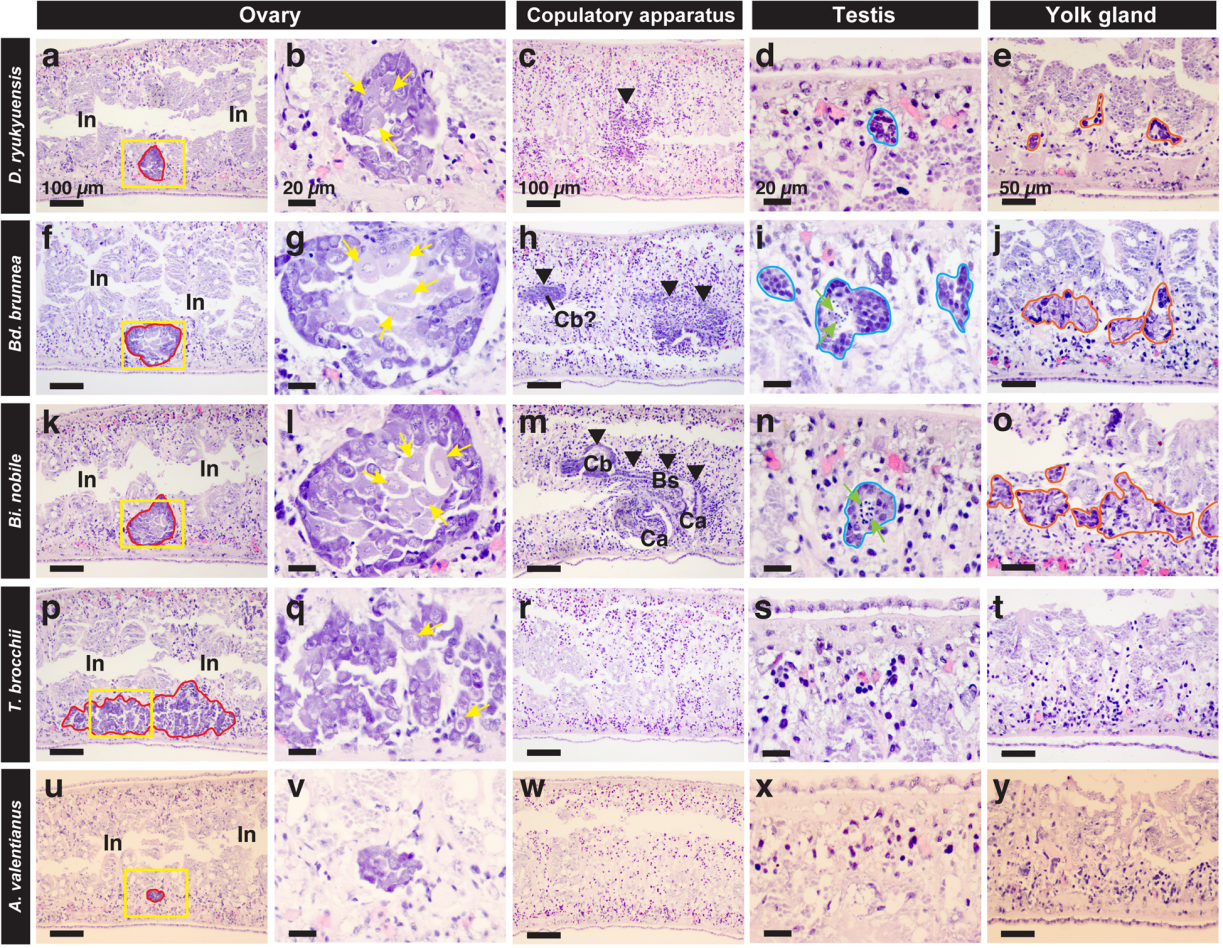
Histological analysis of test worms obtained by the administration of Fr. M0 or M10. Embedded test worms fed with either Fr. M0 or M10 were sagittally sectioned and stained with hematoxylin and eosin (HE). The dorsal sides are at the top. **a**–**e** Fr. M10 from *D. ryukyuensis*, (**f**–**j**) Fr. M10 from *Bd. brunnea*, (**k**–**o**) Fr. M0 from *Bi. nobile,* (**p**–**t**) Fr. M10 from *T. brocchii* and (**u**–**y**) Fr. M0 from *A. valentianus*. (**a**, **f**, **k**, **p**, and **u**) Domains bound by the red line are the female germ cell masses (ovaries). In, intestine. Scale bar, 100 μm. (**b**, **g**, **l**, **q**, and **v**) High magnification of the areas squared by the yellow line in (**a**, **f**, **k**, **p**, and **u**), respectively. Scale bar, 20 μm. (**c**, **h**, **m**, **r**, and **w**) The region indicated by the arrowheads is a developing copulatory apparatus. Ca, common antrum; Cb, copulatory bursa; Bs, bursa stalk. Scale bar, 100 μm. (**d**, **i**, **n**, **s**, and **x)** Domains bounded by the blue line are the male germ cell masses (testes). The cell indicated by the green arrow is a spermatid. Scale bar, 20 μm. (**e**, **j**, **o**, **t**, and **y**) Domains bounded by the orange-colored line are yolk glands. Scale bar, 50 μm

Test worms fed freeze-dried chicken liver homogenate (a vehicle control) did not develop reproductive organs. Concerning the crucial sex-inducing substance contained in the cytosolic fractions, the activity needed for overcoming the point-of-no-return was recognized in both Fr. M0 and M10 from *D. ryukyuensis*, *Bd. brunnea* and *Bi. nobile* (Fig. 4). We also carried out histological examinations of the most sexually mature test worm administered Fr. M0 or M10 from five species (Fig. 5) to confirm the degree of differentiation of reproductive organs, and have summarized the results in Table 1. Worms at stage 4 were obtained by the administration of Fr. M10 from *Bd. brunnea* (Fig. 5f–j), whereas worms at stage 5 were obtained by the administration of Fr. M0 from *Bi. nobile* (Fig. 5k–o). It should be noted that the sex-inducing activity from conspecific sexual worms (Figs. 4 and 5a–e) was significantly weaker than that of *Bd. brunnea* and *Bi. nobile* (Table 2). In particular, the highest sex-inducing activity was found in Fr. M0 from *Bi. nobile* (Figs. 4 and 5, Table 2).

**Table 1:** Summary of the stages by histological changes in sexual induction

Test food		Ovary	Copulatory apparatus	Testis	Yolk gland	Stage of sexual induction
Species	Fraction					
*Dugesia ryukyuensis*	Fr. M10	mature oocyte	primordium	primordium	primordium	Stage 3
*Bdellocephala brunnea*	Fr. M10	mature oocyte	primordium	spermatid	mature tissue	Stage 4
*Bipalium nobile*	Fr. M0	mature oocyte	differentiated	elongating spermatid	mature tissue	Stage 5
*Thysanozoon brocchii*	Fr. M10	immature oocyte	–	–	–	Stage 2
*Ambigolimax valentianus*	Fr. M0	primordium	–	–	–	Stage 1

**Table 2:** Comparative analysis of sex-inducing activity after the point-of-no-return

Test food	Number of worms at stage 3 onwards	Significance^a^	Number of worms at stages 4–5	Significance^b^
Fr. M0				
*Dugesia ryukyuensis*	3 / 30	–	0 / 30	< 0.0002
*Bdellocephala brunnea*	4 / 30	–	0 / 30	< 0.0002
*Bipalium nobile*	30 / 30	< 2.44E-12	13 / 30	–
Fr. M10				
*Dugesia ryukyuensis*	7 / 29	–	0 / 29	< 0.0002
*Bdellocephala brunnea*	20 / 25	< 4.24E-5	2 / 25	< 0.008
*Bipalium nobile*	21 / 30	< 4.21E-4	0 / 30	< 0.0002

^a^Probability was calculated by chi-square test or Fisher’s test and compared with each fraction of *Dugesia ryukyuensis*

^b^Probability was calculated by chi-square test and compared with the Fr. M0 from *Bipalium nobile*

The marine flatworm *T. brocchii* (Polycladida) is a more distant species from *D. ryukyuensis* (Tricladida) than the other two turbellarian species. Although the administration of the Fr. M0, M10, and M100 fractions from *T. brocchii* induced a pair of ovaries externally in a statistically significant number of test worms, these fractions did not contain enough sex-inducing activity to overcome the point-of-no-return (Fig. 4 and Table 3). Histological examination revealed that the administration of Fr. M10 from *T. brocchii* did not induce reproductive organs other than ovaries (Fig. 5p–t). The ovaries developed along the anterior-posterior axis of the worms (Fig. 5p). Since the induced ovaries contained oocytes (Fig. 5q), we inferred that stage 2 worms were obtained by the administration of Fr. M10 from *T. brocchii* (Fig. 3 and Table 1).

**Table 3:** The ovary-inducing activity in the cytosolic fractions from *Thysanozoon brocchii* and *Ambigolimax valentianus*

Test food	Number of worms at stage 1–2	Significance^a^
Chicken liver (Control)	0 / 30	–
*Thysanozoon brocchii*		
Fr. M0	14 / 30	< 7.01E-5
Fr. M10	30 / 30	< 9.49E-15
Fr. M100	10 / 28	< 0.0011
*Ambigolimax valentianus*		
Fr. M0	4 / 30	–
Fr. M10	0 / 30	–
Fr. M100	0 / 29	–

^a^Probability was calculated by chi-square test and compared with the control

**Fig. 6:**
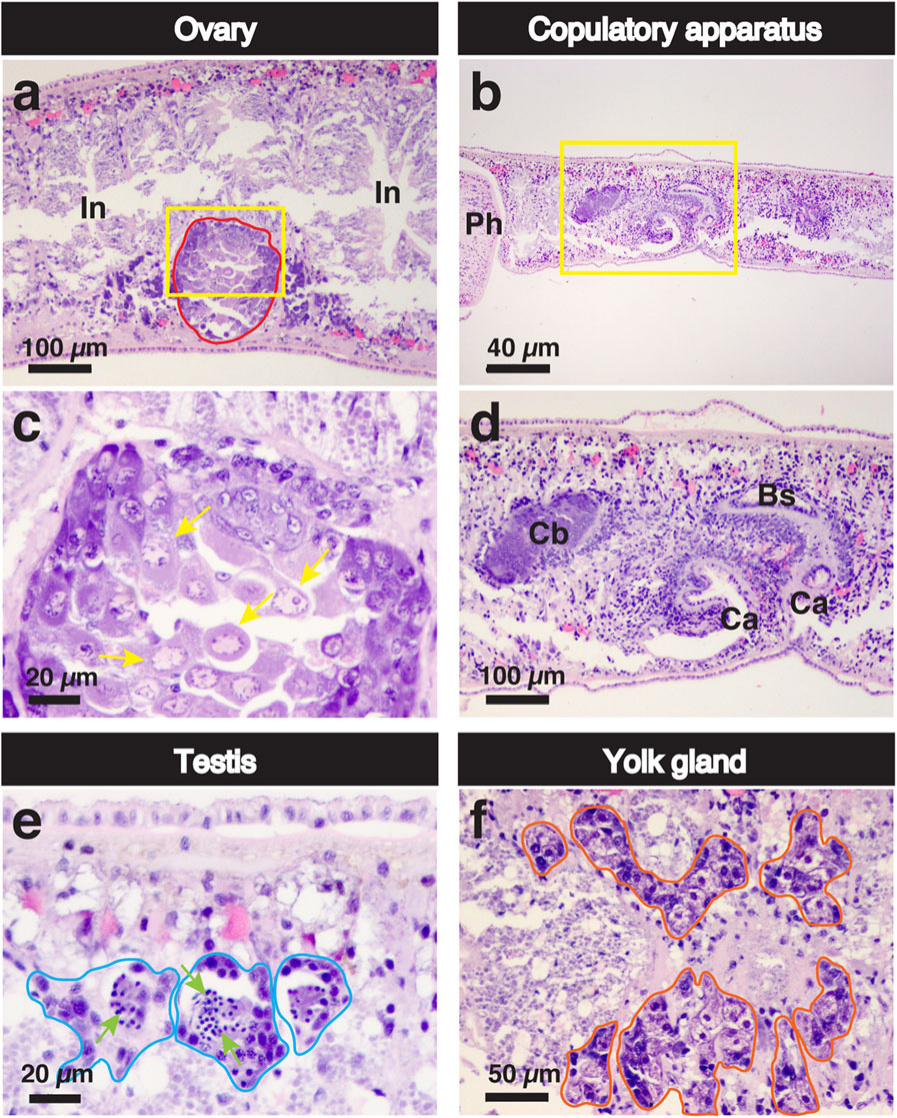
Histological analysis of test worms obtained by the administration of the EtOAc layer of *B. nobile.* Embedded a test worm fed with about ten times the standard dose of the EtOAc layer of *B. nobile* was sagittally sectioned and stained with HE. The dorsal sides are at the top. **a** ovary, **b** copulatory apparatus, **e** testes, and **f** yolk glands. (**c** and **d**) High magnification of the area squared by the yellow line in (**a** and **b**), respectively. Domains bound by the red line in (**a**), the blue line in (**e**), and the orange line in (**f**) are the female germ cell masses (ovaries), the male germ cell masses (testes) and yolk glands, respectively. The cells indicated by the yellow arrow in (**c**) and green arrow in (**e**) are an oocyte and a spermatid, respectively. Ca, common antrum; Cb, copulatory bursa; Bs, bursa stalk; In, intestine; Ph, pharynx

To date, we have focused on only the hydrophilic sex-inducing substance. In the present study, sex-inducing activity in more hydrophobic compounds from the precipitate was carefully examined for the first time, to the best of our knowledge. The EtOAc layers from the four turbellarian species showed only weak ovary-inducing activity in the test worms (Fig. 4). However, about 10 times the amount of the EtOAc layer from *Bi. nobile* had the ability to overcome the point-of-no-return (Fig. 4). Histological examination revealed that worms at stage 5 were obtained by the administration of about ten times the standard dose of the EtOAc layer from *Bi. nobile* (Fig. 6).

The slug, *A. valentianus* (Mollusca), did not have significant sex-inducing activity, although a pair of small ovaries became visible in a few asexual worms of *D. ryukyuensis* in this assay (Figs. 4 and 5, Table 3). In *D. ryukyuensis*, primordial ovaries were histologically identified even in asexual worms, although they were barely visible externally [22] (Fig. 3). The ovarian morphology in the worms fed with the test food containing the Fr. M0 of *A. valentianus* was nearly identical to that of the primordial ovaries (Fig. 5u, v).

### Is the hydrophilic crucial sex-inducing substance a peptide?

**Fig. 7:**
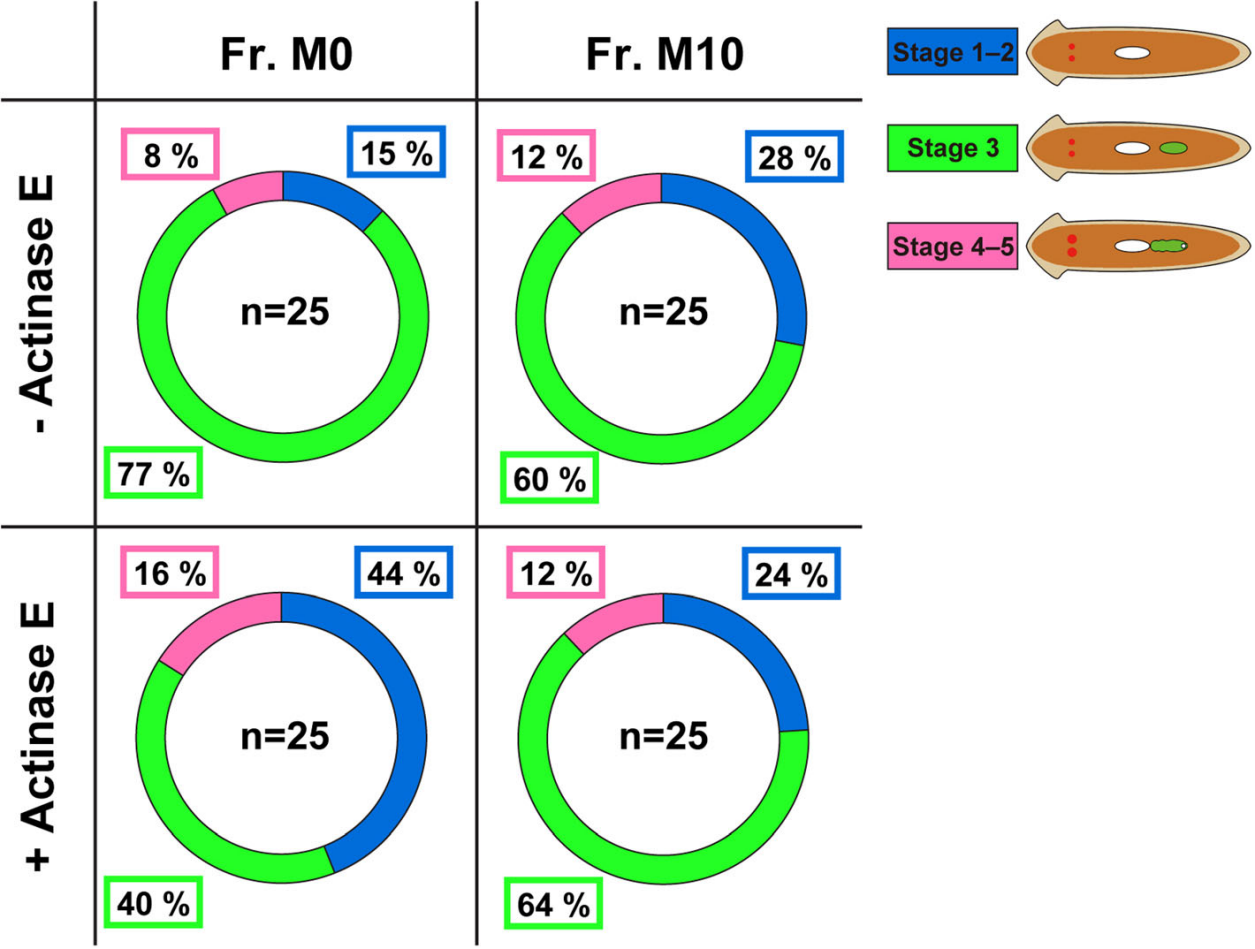
Comparison of sex-inducing activity between + and – Actinase E samples. Test worms at the three stages of sexual induction identified by external observation were expressed as a doughnut chart. The illustrations of worms in the figure correspond to those in Fig. 3b. The number of test worms after the bioassay is shown in the center of a doughnut chart. The percentage of worms at each of the three stages of sexual induction is shown in the box drawn by colored line. There was no significant difference between Fr. M0 + Actinase E and Fr. M0 – Actinase E, and Fr. M10 + Actinase E and Fr. M10 – Actinase E (a chi-square test)

The crucial sex-inducing substance present in the Fr. M0 and the M10 fractions from freshwater planarians *D. ryukyuensis* and *Bd. brunnea* is hydrophilic [26]. Even in a land planarian *Bi. nobile*, strong sex-inducing activity was recovered in Fr. M0 and M10 (Fig. 4). Recently, we found that the crucial sex-inducing substance of Fr. M0 and M10 derived from *Bd. brunnea* is heat-stable [28]. These characteristics do not preclude the possibility that the crucial sex-inducing substance is a peptide. This information is important in terms of the identification of the crucial sex-inducing substance. In the present study, to estimate whether the crucial sex-inducing substance is a peptide, we carried out treatment with Actinase E, a powerful enzyme for the elimination of peptides/proteins, in the Fr. M0 and M10 fractions in *Bd. brunnea.* The sex-inducing activity of Fr. M0 and M10 fractions in *Bd. brunnea* did not decrease, even though these fractions were treated with Actinase E (Fig. 7). This suggested that the crucial sex-inducing substance is not a peptide.

### Feeding experiment with cocoons laid by freshwater planarians

In some orders of turbellarians, worms have yolk glands, a reproductive organ filled with nurse cells; namely, yolk gland cells. Their eggs are ectolecithal (cocoons), which have several fertilized eggs and numerous yolk gland cells [24]. In *D. ryukyuensis* (Tricladida), the existence of intact yolk gland cells in fresh cocoons collected within a day of deposition has been suggested by quantitative reverse transcription polymerase chain reaction analysis of a yolk gland marker gene [23].

**Fig. 8:**
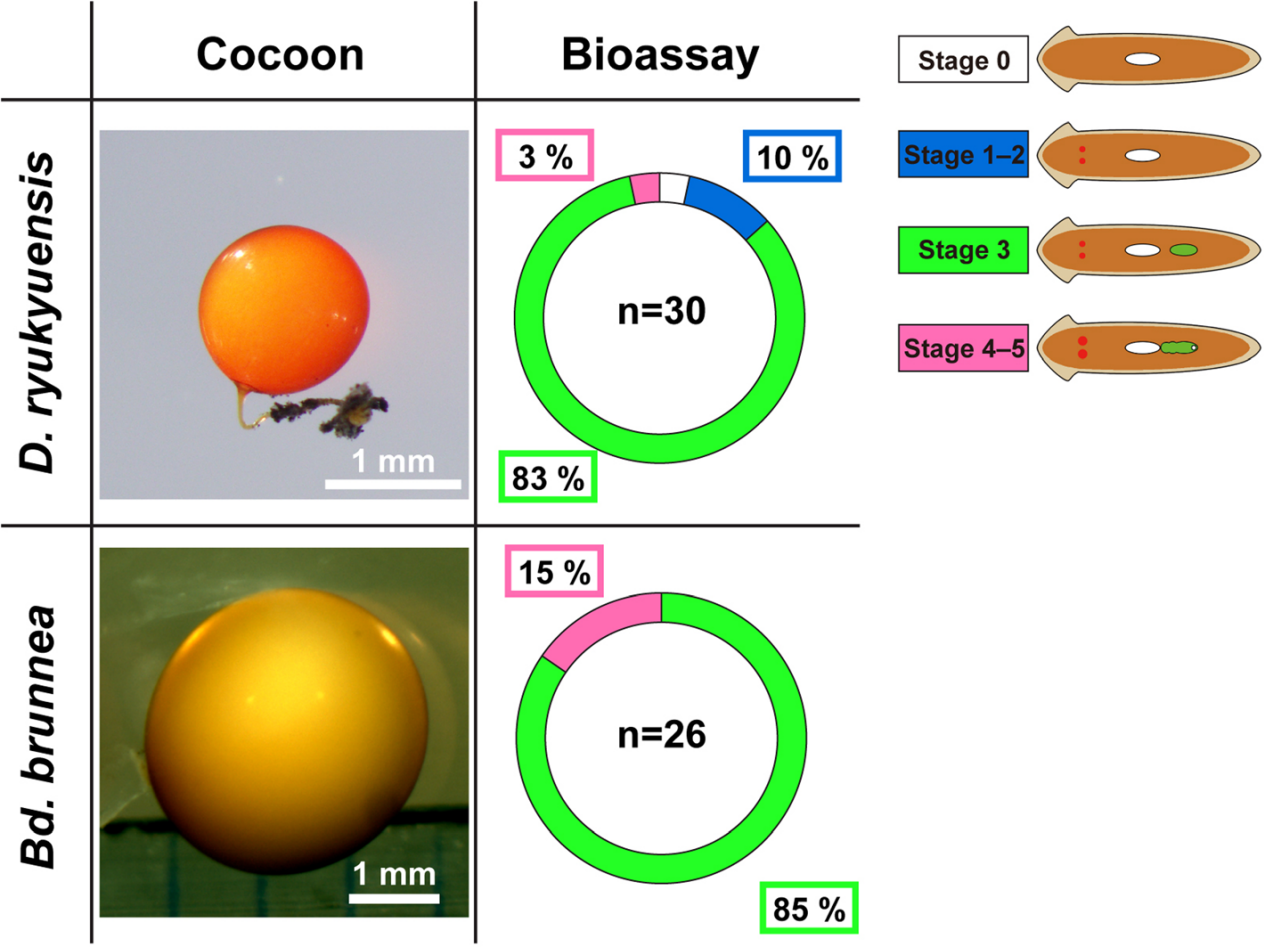
Feeding experiment with cocoons laid by freshwater planarians. Thirty test worms were fed cocoons laid by *Dugesia ryukyuensis* or *Bdellocephala brunnea* daily for 4 weeks. The figure contains two photographs of cocoons laid by *D. ryukyuensis* or *Bd. brunnea.* Test worms at the three stages of sexual induction identified by external observation were expressed as a doughnut chart. The illustrations of worms in the figure correspond to those in Fig. 3b. The number of test worms after the bioassay is shown in the center of a doughnut chart. The percentage of worms at each of the three stages of sexual induction is shown in the box drawn by colored line

The results of a comparative analysis of sex-inducing activity suggest that the crucial sex-inducing substance needed to overcome the point-of-no-return in asexual worms of *D. ryukyuensis* is present in worms, at least in Tricladida but not Polycladida. An anatomically crucial difference between Tricladida and Polycladida is the presence or absence of the yolk glands. This finding led us to examine the sex-inducing activity of yolk glands (cocoons) in freshwater planarians *D. ryukyuensis* and *Bd. brunnea*. As we expected, asexual test worms overcame the point-of-no-return when they were fed cocoons of *D. ryukyuensis* and *Bd. brunnea* daily for 4 weeks (Fig. 8).

## Discussion

Turbellarian species generally have pluripotent stem cells called neoblasts (i.e., Catenulidae [29], Macrostomida [29–31], Polycladida [32]). They undergo homeostatic regulation of their body size by “cell turnover,” which requires neoblasts [33], and have the capacity for regeneration owing to these neoblasts. Furthermore, they can post-embryonically produce germ line cells from neoblasts. Some low-molecular-weight compounds are involved in post-embryonic germ cell development, yet little information about them exists. Owing to these characteristics, some turbellarian species can switch between an asexual and a sexual state in nature. They may use the low-molecular-weight compounds involved in post-embryonic germ cell development as sex-inducing substances when they alternate from an asexual to a sexual state. Thus, sex-inducing substances are important from the aspects of both developmental and reproductive biology.

A feeding assay system using asexual test worms (the OH strain) of the freshwater planarian *D. ryukyuensis* (Tricladida, Continenticola, Dugesiidae) [22] is useful in evaluating sex-inducing activity. In *D. ryukyuensis*, sexual induction has a point-of-no-return between stages 2 and 3 (Fig. 3) [34]. Worms at stages 1 and 2 return to the asexual condition if feeding with a test food containing a sex-inducing substance is stopped. In contrast, worms at stage 3 and beyond keep developing sexual organs even if feeding with the test food is stopped. Recently, we identified the ability of d-Trp to induce stage 2 ovaries in asexual worms of *D. ryukyuensis* [23]. However, d-Trp does not induce the other reproductive organs. The crucial sex-inducing substance required to overcome the point-of-no-return has not yet been identified. Previous studies suggested that the crucial sex-inducing substance is evolutionarily conserved in, at least freshwater planarians (Additional file 1).

In this study, to further estimate a phylogenetic relationship of species containing the crucial sex-inducing substance, a comprehensive comparison of sex-inducing activity in asexual worms of *D. ryukyuensis* was carried out using the freshwater planarians *D. ryukyuensis* and *Bd. brunnea*, land planarian *Bi. nobile* (Tricladida, Continenticola, Bipaliidae), and marine flatworm *T. brocchii* (Polycladida) as sources of the sex-inducing substance. A slug *Ambigolimax valentianus* (Mollusca) was also used.

The present study clearly showed that in the cytosolic fractions, the probability of conspecific worms displaying sex-inducing activity was always lower than that of *Bd. brunnea* and *Bi. nobile* (Table 2). In particular, the Fr. M0 from the land planarian *Bi. nobile* showed the highest sex-inducing activity in the cytosolic fractions among four turbellarian species (Figs. 4 and 5, Tables 1 and 2). Molecular phylogenetic analysis of freshwater and land planarians has suggested that in terms of phylogenetic distance, freshwater planarians in the family Dugesiidae and land planarian in the family Bipaliidae are more closely related than freshwater planarians in the family Dendrocoelidae and those in the family Planariidae [35]. The ability to produce crucial sex-inducing activity in asexual planarians in the family Dugesiidae has been confirmed in sexual planarians of the families Dendrocoelidae, Planariidae, and Dugesiidae (Additional file 1). The ability of the land planarian *Bi. nobile* (Bipaliidae) to produce strong sex-inducing activity in asexual worms of *D. ryukyuensis* (Dugesiidae) may be consistent with the aforementioned phylogenetic relationship.

In contrast, insufficient sex-inducing activity to overcome the point-of-no-return was found in the cytosolic fraction of a marine flatworm *T. brocchii* (Fig. 4), although the induced ovaries were extraordinarily large and contained oocytes (Fig. 5p, q). It was noted that of all the species, only the Fr. M100 of *T. brocchii* showed significant sex-inducing activity (Fig. 4 and Table 3). The marine flatworm *T. brocchii* may possess an analog with the extremely low sex-inducing activity, or only an ovary-inducing substance like d-Trp. Additionally, there is possibly a compound unique to the sex-inducing activity in Fr. M100. In gastropod mollusks containing *A. valentianus*, the tripeptide l-Asn-d-Trp-l-Phe-NH_2_ (NdWFamide) acts as a neuropeptide [36–40]. Thus, *A. valentianus* must contain free d-Trp as a degradant of this neuropeptide. In the fractionation procedure, d-Trp is recovered primarily in Fr. M0 [23]. It may be reasoned that a few asexual worms of *D. ryukyuensis* fed with the test food containing the Fr. M0 of *A. valentianus* developed a pair of ovaries (Figs. 4 and 5u, v). These results suggest that there might be a common compound or a functional analog as the hydrophilic crucial sex-inducing substance in Tricladida, but not in Polycladida.

In the present study, the sex-inducing activity of more hydrophobic compounds recovered in EtOAc layer was examined. The administration of the EtOAc layer in *D. ryukyuensis*, *Bd. brunnea*, and *T. brocchii* induced only a pair of ovaries, even though asexual worms of *D. ryukyuensis* were fed about ten times the standard dose of the EtOAc layer (about 39 mg dry weight) (Fig. 4). This suggests that there is a hydrophobic ovary-inducing substance in these species. However, approximately ten times the dry weight of the EtOAc layer from *Bi. nobile* resulted in enough sex-inducing activity required to overcome the point-of-no-return (Figs. 4 and 6). The existence of a hydrophobic crucial sex-inducing substance in *Bi. nobile* may be associated with terrestrial organisms.

There is much debate on the identity of the organs or tissues responsible for producing the crucial sex-inducing substance. One theory is that the putative hormone produced by the testes is responsible for the development of the copulatory apparatus [41]. Indeed, it was suggested that sexual worms of *D. ryukyuensis* lacking testes after treatment with the RNAi of *Dr-nanos* and *Dr-piwi1* could not maintain their acquired sexuality [42, 43]. The other theory is that they are derived from the neurosecretion responsible for gonad maturation as described above [44–46]. Interestingly, neuropeptide NPY-8 is specifically associated with testicular differentiation in the freshwater planarian *Schmidtea mediterranea* [47]. The RNAi knockdown of *npy-8* in sexually mature worms results in the regression of the testes, which acts to maintain planarian sexuality. However, to date, the yolk gland has not been a candidate for the source of the crucial sex-inducing substance.

Together, the results in the present study suggest that turbellarians possess a compound(s) with the sex-inducing activity in asexual worms of *D. ryukyuensis*. Furthermore, the crucial sex-inducing substance needed to overcome the point-of-no return in asexual worms of *D. ryukyuensis* may be contained in worms of Tricladida, but not those of Polycladida. An anatomically crucial difference between Tricladida and Polycladida is the presence or absence of yolk glands. Immediately after the point-of-no-return (stage 3), primordial yolk glands emerged in *D. ryukyuensis* (Fig. 3). Recently, we also found that a large amount of l-Trp is incorporated and pooled in the yolk glands, resulting in the accumulation of d-Trp that is involved in the ovarian development of asexual worms as a sex-inducing substance [23]. Motivated by these findings, we fed the asexual worms of *D. ryukyuensis* with fresh cocoons of *D. ryukyuensis* and *Bd. brunnea* containing numerous yolk gland cells, resulting in full sexual induction (Fig. 8). Besides, the sex-inducing activity of Fr. M0 and M10 from *Bd. brunnea* did not decrease with treatment with Actinase E, which is a powerful enzyme causing the elimination of peptides/proteins in a solution (Fig. 7). We concluded that the crucial sex-inducing substance in the asexual worms of *D. ryukyuensis* is present in yolk glands and is not a peptide.

A slug, *A. valentianus*, is a food for the land planarian *Bi. nobile* in nature. There were no fractions from *A. valentianus* that produced significant sex-inducing activity in asexual worms of *D. ryukyuensis* (Fig. 4). The crucial sex-inducing substance could be de novo synthesized in the yolk glands of Tricladida. In the present study, we used worms from two orders (Tricladida and Polycladida) in Turbellaria, namely macroturbellarians as sources of a sex-inducing substance. As worms in the other nine orders in Turbellaria, namely microturbellarians, are small, we abandoned using them as sources of a sex-inducing substance. However, six microturbellarians produce ectolecithal eggs (cocoons) like Tricladida [24], meaning that they also have yolk glands (−like organs). They also may contain the crucial sex-inducing substance in the asexual worms of *D. ryukyuensis.* In the near future, we will seek to identify the crucial sex-inducing substance on the basis of the results of the present study.

## Conclusions

Certain low-molecular-weight compounds found in sexually mature animals act as sex-inducing substances during post-embryonic germ cell development when the animals alternate from an asexual to a sexual state (sexual induction). The crucial sex-inducing substance responsible for the sexual induction of freshwater planarians has not yet been identified. An assay system that involves feeding asexual worms of the freshwater planarian *D. ryukyuensis* is useful for evaluating this type of sex-inducing activity. In the present study, to estimate a phylogenetic range of species that may possess compounds with sex-inducing activity in asexual worms of *D. ryukyuensis*, we carried out a comprehensive comparison of the sex-inducing activity containing worms in two orders (Tricladida and Polycladida) in Turbellaria as sources of a sex-inducing substance. Using this assay system, we showed that the three species in Order Tricladida have strong sex-inducing activity and can fully sexualize asexual worms of *D. ryukyuensis*. Interestingly, the sex-inducing activity displayed by the conspecific sexual worms was not higher than that of the freshwater planarian *Bd. brunnea* or land planarian *Bi. nobile*, which belong to the same order. In contrast, the sex-inducing activity displayed by the marine flatworm *T. brocchii*, which belongs to Order Polycladida, was extremely low. On the basis of these results, we found that yolk glands, which exist in Tricladida but not Polycladida, possibly contain the crucial sex-inducing substance (hydrophilic and heat-stable, but not a peptide) that can fully sexualize asexual worms of *D. ryukyuensis*. The results obtained in this study will contribute to the identification of the crucial sex-inducing substance.

## Additional files

Additional file 1: **Table S1.**. Relationship between test worm and food in the experimental sexual induction. (PDF 71 kb)

Additional file 2: **Table S2.** Dry weight of fractions derived from the cytosolic fraction. (PDF 69 kb)

Additional file 3: **Table S3.** Weight of precipitates and EtOAc layers. (PDF 69 kb)

Additional file 4: **Figure S1.** Preparation of foods for the bioassay on Fr. M0 and M10 of *Bd. brunnea* treated with Actinase E. (PDF 303 kb)
