# Correction to: A comprehensive comparison of sex-inducing activity in asexual worms of the planarian Dugesia ryukyuensis: the crucial sex-inducing substance appears to be present in yolk glands in Tricladida

**DOI:** 10.1186/s40851-018-0106-y

**Published:** 2018-08-31

**Authors:** Haruka Nakagawa, Kiyono Sekii, Takanobu Maezawa, Makoto Kitamura, Soichiro Miyashita, Marina Abukawa, Midori Matsumoto, Kazuya Kobayashi

**Affiliations:** 1 Department of Biology, Faculty of Agriculture and Life Science, Hirosaki University, Aomori, Japan; 2 Advanced Science Course, Department of Integrated Science and Technology, National Institute of Technology, Tsuyama College, Okayama, Japan; 3 Center for Integrated Medical Research, School of Medicine, Keio University, Tokyo, Japan; 4 Department of Biosciences and Informatics, Keio University, Yokohama, Japan

## Correction

Following publication of the original article [1] the author advised that unfortunately in the ‘Discussion’ section the letters ‘L’ and ‘D’ in some ‘enantiomer’ expressions were not written in the format of ‘small capitals’. Rather, they were written as lower-case letters.

This formatting is an important nomenclature in chemistry, the author confirmed, advising:

“Enantiomers (mirror-image isomer) of amino acids (L or D) is always expressed by ‘small capitals’ ”

As such, the original article [1] has been corrected to reflect this.
