# Supplementary material for: A comprehensive comparison of sex-inducing activity in asexual worms of the planarian Dugesia ryukyuensis: the crucial sex-inducing substance appears to be present in yolk glands in Tricladida
Source: Zoological Lett. 2018 Jun 12;4:14. doi: 10.1186/s40851-018-0096-9 (PMC5996458; doi:10.1186/s40851-018-0096-9)
Supplement: Supplementary file 1 — Table S1.. Relationship between test worm and food in the experimental sexual induction. (PDF 71 kb) [file 40851_2018_96_MOESM1_ESM.pdf]

Table S1 Relationship between test worm and food in the experimental sexual induction

Asexual freshwater planarians used as test worms (family)	Sexual freshwater planarians used as test foods (family)	References
Dugesia gonocephala (Dugesiidae)	*Polycelis nigra* (Planariidae)	Grasso and Benazzi (1973)
Dugesia gonocephala (Dugesiidae)	*Schmiditea lugubris** (Dugesiidae)	
Dugesia gonocephala (Dugesiidae)	*Dendrocoelum lacteum* (Dendrocoelidae)	Benazzi and Grasso (1977)
Dugesia gonocephala (Dugesiidae)	*Girardia anceps** (Dugesiidae)	
Dugesia gonocephala (Dugesiidae)	*Girardia dorotoceplala** (Dugesiidae)	
Dugesia gonocephala (Dugesiidae)	*Polycelis nigra* (Planariidae)	
Dugesia gonocephala (Dugesiidae)	*Schmiditea lugubris** (Dugesiidae)	
*Girardia tigrina** (Dugesiidae)	*Polycelis nigra* (Planariidae)	
Dugesia anderlani (Dugesiidae)	*Girardia tigrina** (Dugesiidae)	Hauser (1987)
*Dugesia japonica** (Dugesiidae)	*Bdellocephala brunnea* (Dendrocoelidae)	Sakurai (1981)
*Dugesia japonica** (Dugesiidae)	*Dugesia japonica** (Dugesiidae)	
Dugesia japonica (Dugesiidae)	*Dugesia japonica* (Dugesiidae)	Teshirogi (1986)
Dugesia japonica (Dugesiidae)	*Bdellocephala brunnea* (Dendrocoelidae)	
Dugesia japonica (Dugesiidae)	*Polycelis sappro* (Planariidae)	
Dugesia ryukyuensis (Dugesiidae)	*Bdellocephala brunnea* (Dendrocoelidae)	Kobayashi et al. (1999)
Dugesia ryukyuensis (Dugesiidae)	*Dugesia ryukyuensis* (Dugesiidae)	Kobayashi et al. (2002)

* Present scientific name is shown.
