# Supplementary material for: A comprehensive comparison of sex-inducing activity in asexual worms of the planarian Dugesia ryukyuensis: the crucial sex-inducing substance appears to be present in yolk glands in Tricladida
Source: Zoological Lett. 2018 Jun 12;4:14. doi: 10.1186/s40851-018-0096-9 (PMC5996458; doi:10.1186/s40851-018-0096-9)
Supplement: Supplementary file 2 — Table S2. Dry weight of fractions derived from the cytosolic fraction. (PDF 69 kb) [file 40851_2018_96_MOESM2_ESM.pdf]

Table S2 Dry weight of fractions derived from the cytosolic fraction

Species	Fr. M0 (mg)	Fr. M10 (mg)	Fr. M100 (mg)
D. ryukyuensis	21.7	4.2	5.0
Bd. brunnea	16.6	3.9	8.1
Bi. nobile	25.4	7.9	14.3
T. brocchii	26.9	5.5	6.7
A. valentianus	35.3	7.5	11.5
