# Supplementary material for: A comprehensive comparison of sex-inducing activity in asexual worms of the planarian Dugesia ryukyuensis: the crucial sex-inducing substance appears to be present in yolk glands in Tricladida
Source: Zoological Lett. 2018 Jun 12;4:14. doi: 10.1186/s40851-018-0096-9 (PMC5996458; doi:10.1186/s40851-018-0096-9)
Supplement: Supplementary file 3 — Table S3. Weight of precipitates and EtOAc layers. (PDF 69 kb) [file 40851_2018_96_MOESM3_ESM.pdf]

Table S3 Weight of precipitates and EtOAc layers

Species	Precipitate (g in wet weight)	EtOAc layer (mg in dry weight)
D. ryukyuensis	2.2638	52.7
Bd. brunnea	2.2652	42.0
Bi. nobile	3.0324	41.0
T. brocchii	1.4861	34.9
A. valentianus	1.7738	63.8
