# Supplementary figures and images for: A comprehensive comparison of sex-inducing activity in asexual worms of the planarian Dugesia ryukyuensis: the crucial sex-inducing substance appears to be present in yolk glands in Tricladida
Source: Zoological Lett. 2018 Jun 12;4:14. doi: 10.1186/s40851-018-0096-9 (PMC5996458; doi:10.1186/s40851-018-0096-9)

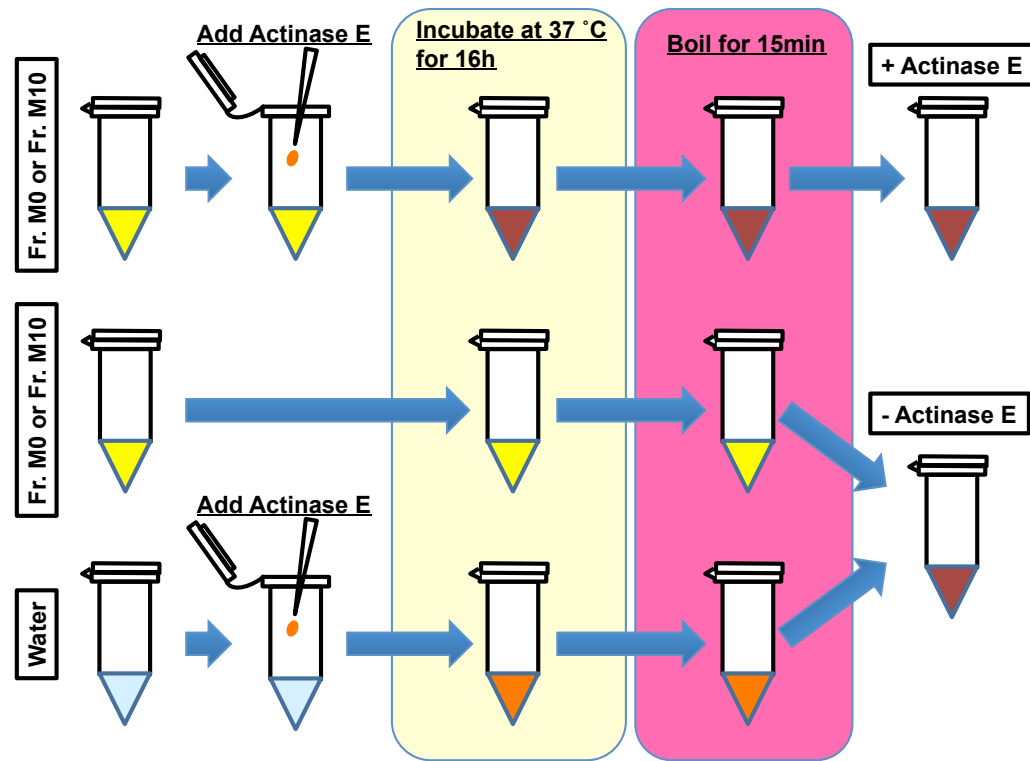

Supplement: Supplementary file 4 — Figure S1. Preparation of foods for the bioassay on Fr. M0 and M10 of Bd. brunnea treated with Actinase E. (PDF 303 kb) [file 40851_2018_96_MOESM4_ESM.pdf]
